# Development and Validation of a Deep Learning Algorithm for Differentiation of Choroidal Nevi from Small Melanoma in Fundus Photographs

**DOI:** 10.1016/j.xops.2024.100613

**Published:** 2024-08-30

**Authors:** Shiva Sabazade, Marco A. Lumia Michalski, Jakub Bartoszek, Maria Fili, Mats Holmström, Gustav Stålhammar

**Affiliations:** 1 Division of Eye and Vision, Department of Clinical Neuroscience, Karolinska Institutet, Stockholm, Sweden; 2 Ocular Oncology Service, St. Erik Eye Hospital, Stockholm, Sweden; 3 Department of Ophthalmology, Kalmar County Hospital, Kalmar, Sweden; 4 Expligence, Stockholm, Sweden; 5 St. Erik Ophthalmic Pathology Laboratory, St. Erik Eye Hospital, Stockholm, Sweden

**Keywords:** Artificial intelligence, Choroidal nevi, Deep learning, Melanoma, Uveal melanoma

## Abstract

**Purpose:**

To develop and validate a deep learning algorithm capable of differentiating small choroidal melanomas from nevi.

**Design:**

Retrospective multicenter cohort study.

**Participants:**

A total of 802 images from 688 patients diagnosed with choroidal nevi or melanoma.

**Methods:**

Wide field and standard field fundus photographs were collected from patients diagnosed with choroidal nevi or melanoma by ocular oncologists during clinical examinations. A lesion was classified as a nevus if it was followed for at least 5 years without being rediagnosed as melanoma. A neural network optimized for image classification was trained and validated on cohorts of 495 and 168 images and subsequently tested on independent sets of 86 and 53 images.

**Main Outcome Measures:**

Area under the curve (AUC) in receiver operating characteristic analysis for differentiating small choroidal melanomas from nevi.

**Results:**

The algorithm achieved an AUC of 0.88 in both test cohorts, outperforming ophthalmologists using the Mushroom shape, Orange pigment, Large size, Enlargement, and Subretinal fluid (AUC 0.77) and To Find Small Ocular Melanoma Using Helpful Hints Daily (AUC 0.67) risk factors (DeLong’s test, *P* < 0.001). The algorithm performed equally well for wide field and standard field photos (AUC 0.89 for both when analyzed separately). Using an optimal operating point of 0.63 (on a scale from 0.00 to 1.00) determined from the training and validation datasets, the algorithm achieved 100% sensitivity and 74% specificity in the first test cohort (F-score 0.72), and 80% sensitivity and 81% specificity in the second (F-score 0.71), which consisted of images from external clinics nationwide. It outperformed 12 ophthalmologists in sensitivity (Mann–Whitney *U*, *P* = 0.006) but not specificity (*P* = 0.54). The algorithm showed higher sensitivity than both resident and consultant ophthalmologists (Dunn's test, *P* = 0.04 and *P* = 0.006, respectively) but not ocular oncologists (*P* > 0.99, all *P* values Bonferroni corrected).

**Conclusions:**

This study develops and validates a deep learning algorithm for differentiating small choroidal melanomas from nevi, matching or surpassing the discriminatory performance of experienced human ophthalmologists. Further research will aim to validate its utility in clinical settings.

**Financial Disclosure(s):**

Financial DisclosuresProprietary or commercial disclosure may be found in the Footnotes and Disclosures at the end of this article.

Choroidal nevi are present in 4% to 7% of Caucasian adults and in <1% of individuals of African and Asian descent.^1^^,^^2^ In contrast, uveal melanomas, while rare, are associated with a high risk for metastatic death.^3^^,^^4^ Separating the 2 can be a considerable challenge.

It has been estimated that the risk of malignant transformation of choroidal nevi is 0.2%.^5^ Risk factors include orange pigment, subretinal fluid, dome-shape, low internal reflectivity on ultrasonography, and increasing size.^6^^,^^7^ Observation for growth is often employed to differentiate benign from malignant lesions, and as a reason for referral to specialized centers.^8^ However, even small tumors can be deadly. The 15-year competing-risk incidence of metastatic death is 10% for the smallest American Joint Committee on Cancer size category, and a previous multicenter study of 45 patients with small choroidal melanomas highlighted that tumors as small as 3 mm in diameter and 1 mm in thickness at the time of treatment may lead to metastatic death.^9^^,^^10^

Small choroidal melanomas can closely resemble nevi, and lesions that were once small and seemingly indolent may suddenly transition to growth. The challenge of distinguishing low-risk benign lesions from early stage malignancies is considerable, even for experienced ocular oncologists. This difficulty is exacerbated in healthcare systems constrained by a scarcity of subspecialists to oversee such conditions. As a result, many lesions are detected and monitored by ophthalmologists with limited experience. Nevertheless, the importance of promptly differentiating between benign choroidal nevi and malignant melanomas cannot be overstated. Early and accurate identification of melanoma is essential for enhancing patient outcomes through timely intervention. Moreover, precise diagnosis mitigates the risk of unnecessary treatment of benign nevi, thereby protecting patients from interventions that could detrimentally affect their vision and health. Therefore, the development of dependable tools to aid in this challenging task would be valuable.

In this context, the current study seeks to develop and validate a deep learning algorithm designed to differentiate between choroidal nevi and small melanomas using standard and wide field fundus imagery. The definitive classification of these lesions has been previously established by subspecialized ocular oncologists through extensive clinical evaluations employing a comprehensive array of diagnostic instruments. The effectiveness of this algorithm will be assessed in comparison to the diagnostic capabilities of human observers, including ophthalmology residents, consultant ophthalmologists, and subspecialized ocular oncologists.

## Methods

### Aim of the Study

The aim of this study was to develop and validate a deep learning algorithm capable of distinguishing small choroidal melanomas from nevi in both wide field and standard field fundus photographs.

### Datasets

**Figure 1:**
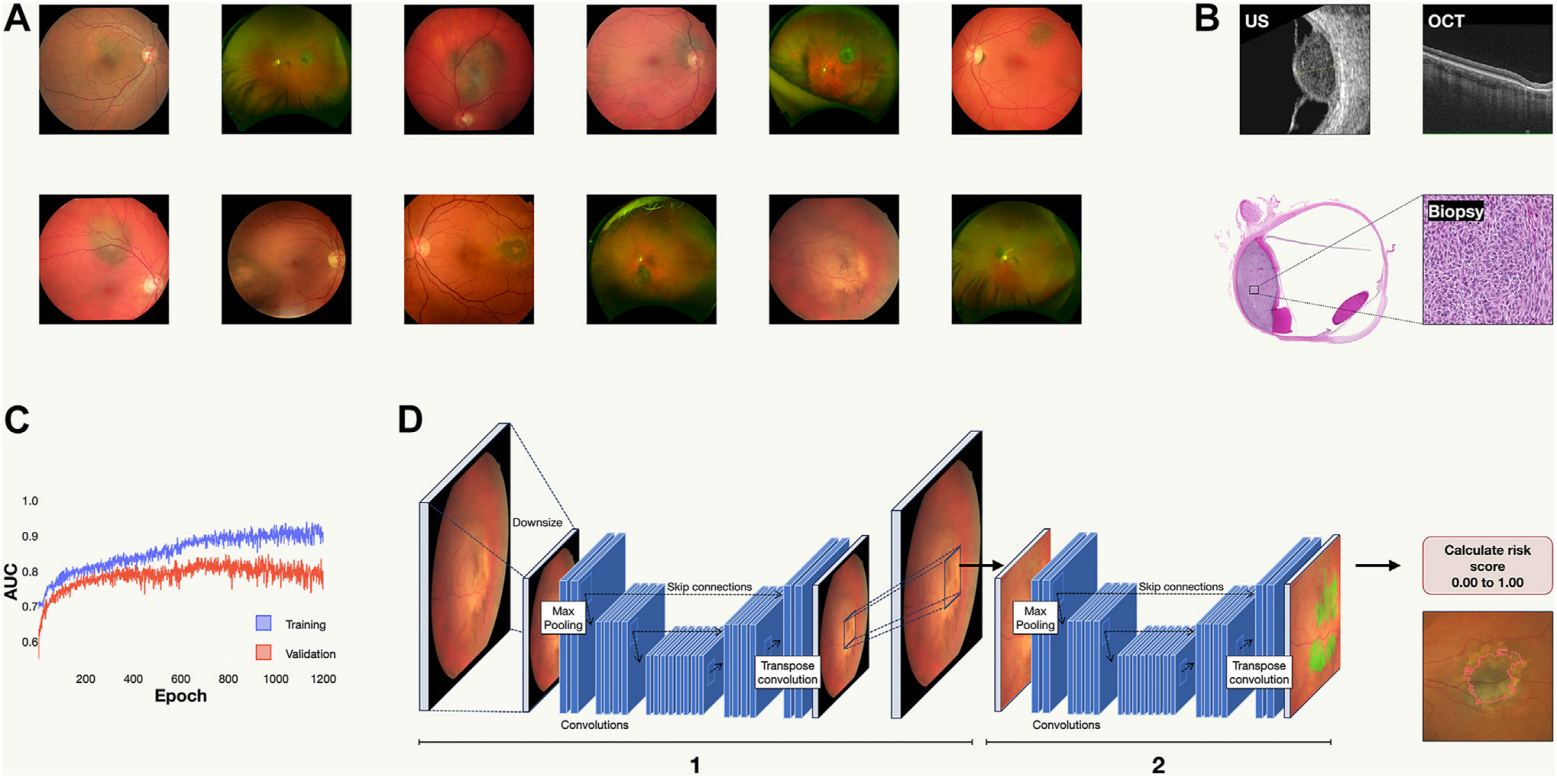
Development, validation, and testing process of the deep learning algorithm for distinguishing small choroidal melanomas from nevi using retinal fundus photographs. **A,** Collection phase: Fundus photographs of choroidal nevi and small melanomas were obtained from multiple institutions, utilizing both ultra-widefield and standard field retinal cameras to ensure the algorithm's applicability to various imaging modalities. **B,** Diagnosis verification: The initial distinction between nevi and melanoma was performed by ocular oncologists through clinical examinations, employing a comprehensive diagnostic approach that included ultrasound, OCT, and biopsies in select cases where necessary. **C,** Algorithm Evaluation: The area under the curve (AUC) served as the primary metric for evaluating the algorithm's performance. The highest melanoma probability pixel within a segmented area was used to calculate the AUC. The network iteration with the peak performance was recorded at epoch 1189, achieving an AUC of 83.4%. **D,** Classification enhancement: To further refine the algorithm, a shallow random forest classifier was trained on the probability outputs, with melanoma images receiving a tenfold increase in weight. This approach was designed to enhance specificity, reduce the incidence of false negatives, and fully utilize the predictive data, beyond merely the highest probability pixel. This modification resulted in an improved AUC of 88.5% in the validation dataset. 1, U-Net 1 identifies the area of interest. 2, U-Net 2 assesses the melanoma risk score, integrating the findings into a comprehensive risk assessment. US = ultrasound.

Fundus photographs were collected from the Ocular Oncology Service at St. Erik Eye Hospital, Stockholm, Sweden, the sole national referral center receiving patients and images from multiple institutions across the country. The photographs were taken using either an ultra-widefield camera (pseudo color images covering 200° of the fundus, Optos, Inc) or a standard field retinal camera (covering 45° of the fundus, Canon Medical Systems Europe, B.V., examples of the collected images are provided in Fig 1A). The collection prioritized small pigmented choroidal lesions, excluding large melanomas as they are relatively easy to distinguish from nevi. Inclusion criteria were as follows:

1. Photograph taken after January 1, 2010, marking a period in which medical records were digitalized which facilitated control over follow-up.
2. Diagnosis of either choroidal melanoma (*International Classification of Diseases, 10th revision* C69.3) or choroidal nevi (*International Classification of Diseases, 10th revision* D31.3).
3. Diagnoses had to be established by a subspecialized ocular oncologist.
4. For lesions diagnosed as nevi at the time of photography, there had to be at least 5 years of follow-up without rediagnosis as a melanoma. Lesions that were diagnosed melanoma at a later point in time (e.g., due to growth) were considered melanomas in this study. This criterion was introduced to facilitate the algorithms detection of early signs of malignancy at a time when a small melanoma is hard to distinguish from a nevus.

Exclusion criteria were:

1. Photographs of low quality, where issues such as focus, movement artifacts, overexposure or underexposure, and reflections hindered the determination of lesion extent or the presence of features like orange pigment or drusen. Minor issues that did not impede assessment, such as blur in smaller areas or focal overexposure, were not sufficient for exclusion.
2. Photographs where our assessment determined that less than half of the lesion was visible, acknowledging the limitation in precisely estimating the size of the portion not visible in the photograph.
3. Lesion obscured by retinal detachment, vitreous bleeding or similar.

Out of 916 images evaluated, 112 were excluded based on the above criteria. An additional 2 images were excluded due to containing sensitive personal information (patient names and personal identification numbers), leaving 802 images for the study. These images were randomized into a training cohort (n = 495), a validation cohort (n = 168), and a test cohort (n = 86). Additionally, 53 images taken in other clinics across the country were reserved for an external test cohort. No image was used in more than 1 cohort and patient level splits were maintained between the cohorts. For each image in the training and validation sets, a mask of the nevus was created, and each image was labeled with the diagnosis (melanoma or nevus). The study was approved by the Swedish Ethical Review Authority (reference 2022-06210-02) and adhered to the tenets of the Declaration of Helsinki. The requirement for informed consent was waived because of the study's retrospective nature, relying solely on previously collected data, including clinical records and images. This research did not involve any new treatments, interventions, tests, analysis of biological samples, or collection of additional sensitive information. Additionally, we followed the Consolidated Reporting Guidelines for Prognostic and Diagnostic Machine Learning Modeling Studies, details of which are provided in a supplementary file (available at www.ophthalmologyscience.org).^11^

### Clinical Diagnosis of Nevi and Melanomas

St. Erik Eye Hospital in Stockholm holds the national responsibility for diagnosing uveal melanoma. Although ophthalmologists from other Swedish institutions may detect potential choroidal tumors and refer patients to our center, a definitive diagnosis of uveal melanoma is made only after a comprehensive examination at our facility. We conduct a comprehensive review of each patient’s medical history, including previous diagnoses, current medication regimens, and records of past ocular examinations. Our diagnostic protocol encompasses a range of procedures: assessment of best-corrected visual acuity and intraocular pressures; wide or standard field fundus photographs with autofluorescence; OCT; slit-lamp biomicroscopy; and A- and B-scan ultrasonography. After this evaluation, we are able to confirm a diagnosis of uveal melanoma in the vast majority of cases. On the rare occasion where clinical examinations are inconclusive, we perform either transvitreal or transscleral biopsies (Fig 1B).^12^^,^^13^ Consequently, all 688 patients in this study, whether diagnosed with a nevus or melanoma, underwent a multimodal examination by an ocular oncologist. This includes the 53 patients in the external test cohort, whose photographs were taken at their home clinics before visiting St. Erik Eye Hospital.

For this study, lesions were also assessed using the Mushroom shape, Orange pigment, Large size, Enlargement, and Subretinal fluid (MOLES) and To Find Small Ocular Melanoma Using Helpful Hints Daily (TFSOM-UHHD) criteria.^14^^,^^15^ The MOLES criteria assigns a score of 0, 1, or 2 for the well-established predictors of Mushroom shape, Orange pigment, Large size, Enlarging tumor, and Subretinal fluid, based on their absence, borderline presence, or presence. Lesions are classified as common nevi, low-risk nevi, high-risk nevi, or probable melanoma, based on their total score being 0, 1, 2, or > 2, respectively. The TFSOM-UHHD criteria stands for “To Find Small Ocular Melanoma Using Helpful Hints Daily,” or Thickness >2 mm, presence of subretinal Fluid, Symptoms, Orange pigment, tumor Margin within 3 mm of the optic disc, Ultrasonographic Hollowness, and the absence of Halo and Drusen. Lesions exhibiting none of these factors have a 3% likelihood of growth over 5 years, suggesting they are most likely choroidal nevi. Those displaying one factor have a 38% chance of growth, while lesions with ≥2 factors have a growth probability >50% at 5 years.^16^

### Data Preprocessing and Model Architecture

In the preprocessing stage, each fundus photograph was resized to a resolution of 1024 × 1536 pixels and adjusted to include 3 channels (red, green, and blue). The wide field images used in this study are generated using red and green lasers, but the resulting images are pseudo-colorized to include the blue spectrum. Images were not cropped. To standardize brightness across the dataset, we normalized the images based on the average brightness of the training dataset, scaling the pixel values to a range of [0, 1].

We implemented a U-net architecture for our model, characterized by 3 down sampling layers and 8 base filters, employing the Rectified Linear Unit as the activation function.^17^ This model was specifically designed to perform as a segmentation tool, with its output subsequently applied to the task of classification. The rationale behind opting for a segmentation approach, as opposed to a direct classification framework, lies in the enhanced interpretability it offers; it allows for clearer visualization of which pixels are being activated by the network. Additionally, by segmenting nevi and melanomas, we provide the network with more detailed information during the training phase, potentially improving the model's learning efficiency and accuracy.

### Model Training

Our model training process utilized 2 distinct U-net models within the Expligence’s Explipipe training framework: one aimed at identifying the area of the lesion and another tasked with classifying whether the lesion is a melanoma. Both models underwent augmentation for brightness and rotation to enhance their robustness.

For the first model, which focuses on detecting the nevus area, categorical cross-entropy served both as the loss function and the evaluation metric. The process involved calculating the weighted central point of the model's output. Subsequently, a bounding box of dimensions 488 × 488 pixels was centered around this point, which then served as the input for the second network.

The second model, designed for melanoma classification, also utilized categorical cross-entropy as its loss function. However, the area under the curve (AUC) was the chosen evaluation metric to identify the most effective network iteration. For AUC calculation, the pixel exhibiting the highest melanoma probability within the segmentation was considered the output. During the training of this second network, only melanoma segmentation masks were used, whereas nevus images were paired with empty segmentation masks. The network achieving the highest AUC was at epoch 1189, with an AUC score of 83.4% (Fig 1C).

In the final step, a shallow random forest classifier was trained on the sorted probability outputs, with a weighting factor of 10 applied to melanoma images. This strategy aimed to increase specificity and minimize false negatives while leveraging the entire output data, rather than focusing solely on the pixel with the highest probability. Implementing this method increased the AUC to 88.5% on the validation set, with an optimal decision threshold of 0.63 (on a 0.00–1.00 scale, Fig 1D).

### Validation of the Algorithm

#### Statistical Analysis and Performance Comparison

Statistical analysis was performed to compare the sensitivities and specificities of human observers (resident ophthalmologists, n = 6; consultant ophthalmologists, n = 3; and ocular oncologists, n = 3) against the gold standard diagnoses of choroidal melanoma or nevi. During the testing phase of fundus photograph assessment, both human evaluators and the algorithm were blinded to any additional patient and lesion information, encompassing clinical diagnoses and follow-up histories. The Kruskal–Wallis test was utilized to assess the overall differences among the groups for both sensitivity and specificity. Post hoc pairwise comparisons were conducted using Dunn test, with Bonferroni correction of *P* values. Mann–Whitney *U* tests were employed to compare the algorithm’s performance with the aggregated sensitivities and specificities of human observers. The AUC of the algorithm was compared with the AUCs of MOLES and TFSOM-UHHD score, and pairwise DeLong’s tests. F-scores were compared using scores above the determined threshold for the algorithm and a MOLES score of ≥3 as indicative of melanoma.^7^ Bonferroni correction was applied to multiple comparisons. *P* values <0.05 were considered to indicate statistical significance, with all *P* values being 2-sided. Statistical significance and confidence intervals were calculated using SciPy (version 0.15.1) and R (R Foundation for Statistical Computing, version 4.2.2) with the stats, PMCMRplus, pROC, dunn.test, caret, and dplyr packages.

## Results

### Descriptive Statistics

In this study, we analyzed 802 images from 688 patients, with 583 images (73%) of lesions classified as nevi and 219 images (27%) classified as melanomas. Among the 688 patients, 382 (56%) were female and 306 (44%) were male. Of nevi and melanoma, 432 (74%) and 129 (59%) had a thickness of 2.0 mm or less, respectively. The distribution of patient and lesion characteristics across the training (n = 495), validation (n = 168), test (n = 86), and external test (n = 53) cohorts is detailed in Table 1.

**Table 1:** Characteristics of Included Patients and Lesions across 3 Cohorts

	Training Cohort	Validation Cohort	Test Cohort	External Test Cohort
*n* images	495	168	86	53
*n* unique patients	410	139	86	53
Sex, *n* (%)				
Male	179 (44)	59 (42)	38 (44)	30 (57)
Female	231 (56)	80 (58)	48 (56)	23 (43)
Age at first observation, mean yrs (SD)	59 (17)	57 (14)	58 (14)	66 (13)
Fundus photography type, *n* (%)				
Wide field	137 (28)	27 (16)	31 (36)	8 (15)
Standard field	358 (72)	141 (84)	55 (64)	45 (85)
Nevi, *n* (%)	367 (74)	123 (73)	57 (66)	36 (68)
Melanomas, *n* (%)	128 (26)	45 (27)	29 (34)	17 (32)
Nevus LBD, *n* (%)				
≤4.5 mm	246 (67)	83 (67)	42 (74)	31 (86)
4.6–6.0 mm	27 (7)	5 (4)	2 (4)	5 (14)
>6.0 mm	94 (26)	35 (29)	13 (23)	0 (0)
Nevus thickness, *n* (%)				
≤2.0 mm	259 (71)	96 (78)	41 (72)	36 (100)
>2.0 mm	17 (4)	5 (4)	2 (4)	0 (0)
N/a	91 (25)	22 (18)	14 (25)	0 (0)
Melanoma LBD, *n* (%)				
≤4.5 mm	33 (26)	14 (31)	6 (21)	0 (0)
4.6–6.0 mm	26 (20)	7 (16)	8 (27)	4 (24)
>6.0 mm	69 (54)	24 (53)	15 (52)	13 (76)
Melanoma thickness, *n* (%)				
≤2.0 mm	74 (58)	33 (73)	12 (41)	10 (59)
>2.0 mm	50 (39)	11 (25)	17 (59)	7 (41)
N/a	4 (3)	1 (2)	0 (0)	0 (0)
Mean MOLES score (SD)				
Overall	1.7 (2.2)	1.6 (2.1)	1.6 (2.2)	2.0 (2.7)
For nevi	0.8 (1.4)	0.9 (1.2)	0.7 (1.2)	0.2 (0.5)
For melanomas	4.3 (2.4)	3.9 (2.5)	3.9 (2.4)	5.8 (1.3)
Mean no. of risk factors TFSOM-UHHD (SD)				
Overall	1.0 (1.1)	1.2 (1.2)	1.2 (1.4)	2.3 (1.7)
For nevi	0.8 (0.8)	0.8 (0.8)	0.8 (1.0)	1.4 (0.7)
For melanomas	1.9 (1.4)	2.5 (1.3)	2.0 (1.8)	4.2 (1.5)

LBD = largest basal diameter; MOLES = Mushroom shape, Orange pigment, Large size, Enlargement, and Subretinal fluid; SD = standard deviation; TFSOM-UHHD = To Find Small Ocular Melanoma Using Helpful Hints Daily.

MOLES assigns a score of 0, 1, or 2 for Mushroom shape, Orange pigment, Large size, Enlarging tumor, and Subretinal fluid, based on their absence, borderline presence, or presence. Lesions are classified as common nevi, low-risk nevi, high-risk nevi, or probable melanoma, based on their total score being 0, 1, 2, or >2, respectively, as described by Roelofs et al 2020. TFSOM-UHHD, stands for “To Find Small Ocular Melanoma Using Helpful Hints Daily,” or Thickness >2 mm, presence of subretinal Fluid, Symptoms, Orange pigment, tumor Margin within 3 mm of the optic disc, Ultrasonographic Hollowness, and the absence of Halo and Drusen, as described by Shields et al 2009.

### Test Cohort Analysis of Sensitivity

**Figure 2:**
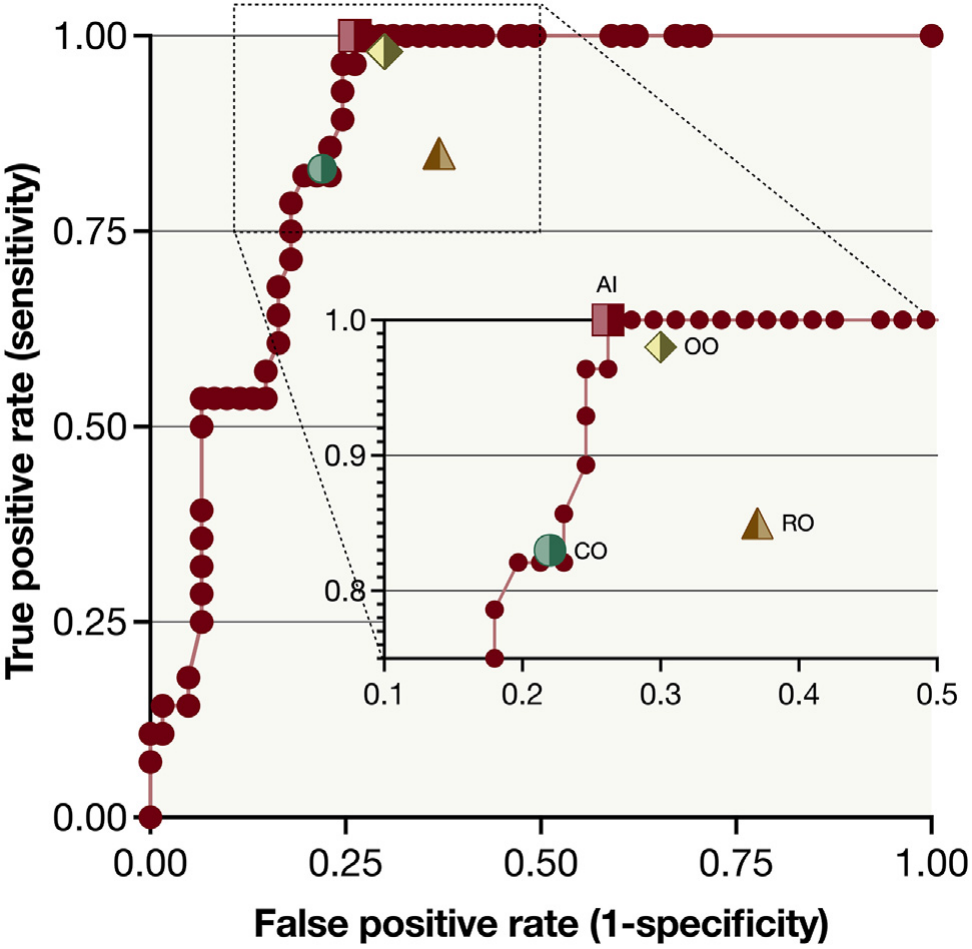
Receiver operating characteristics curves for differentiating small choroidal melanomas from nevi. At a decision threshold of 0.63 (on a 0.00–1.00 scale), the algorithm (AI) reached 100% sensitivity and 74% specificity in the test cohort, indicated by a red box. Resident ophthalmologists (n = 6) assessed the same images with a sensitivity and specificity of 85% and 63%, respectively (brown triangle). Consultant ophthalmologists (n = 3) achieved 83% sensitivity and 78% specificity (green circle), while ocular oncologists (n = 3) achieved 98% sensitivity and 70% specificity (yellow rhombus). The insert narrows the data to a sensitivity range of 75% to 100% and 1-specificity range of 10% to 50%. CO = consultant ophthalmologists; OO = ocular oncologists; RO = resident ophthalmologists.

At the 0.63 decision threshold, the algorithm achieved a sensitivity of 100% and a specificity of 74% for differentiating small choroidal melanomas from nevi in the test cohort of 86 images (Fig 2). The Kruskal–Wallis test revealed a significant difference in sensitivities across the 4 observer categories (algorithm, resident ophthalmologists, consultant ophthalmologists, and ocular oncologists; chi-square statistic (χ^2^): 20.6; *P* < 0.001).

**Figure 3:**
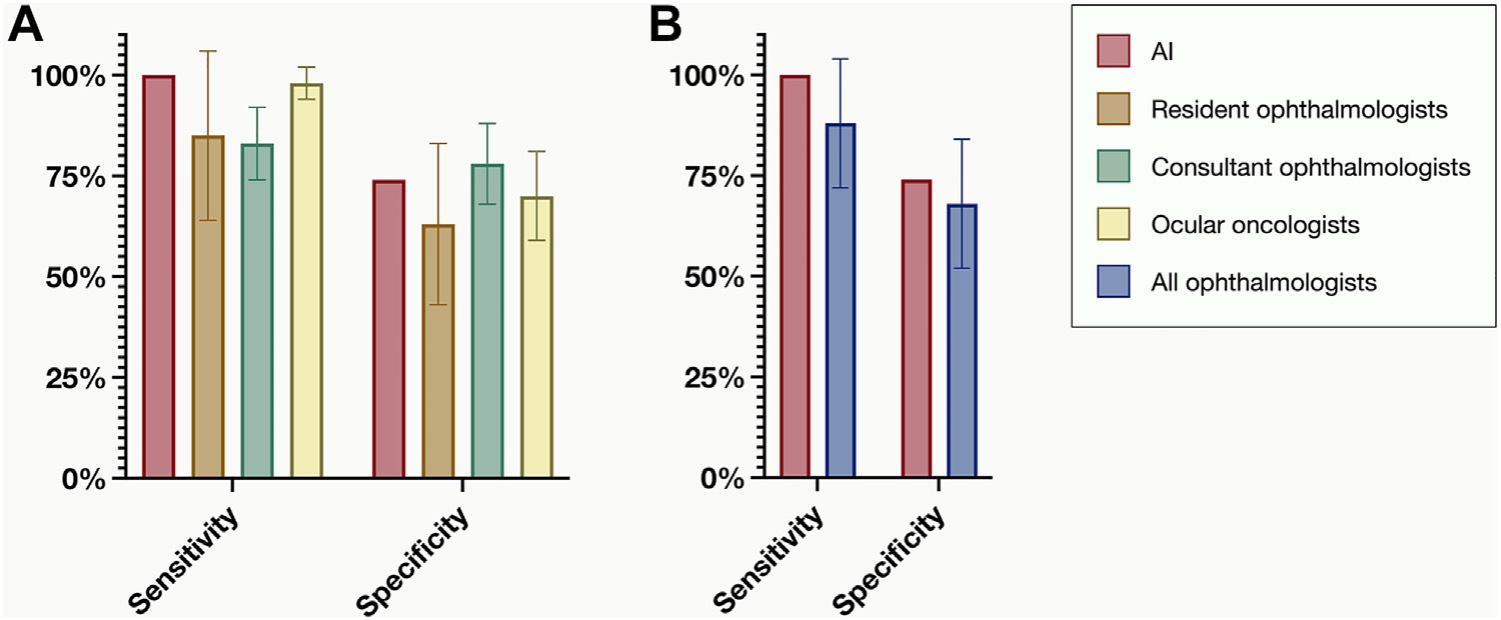
Sensitivity and specificity data presented as bar plots. **A,** Sensitivity and specificity for the algorithm (red), resident ophthalmologists (brown), consultant ophthalmologists (green), and ocular oncologists (yellow). Post hoc pairwise comparisons using Dunn's test revealed that the algorithm exhibited significantly higher sensitivity compared with resident ophthalmologists (100% vs. 85%; *P* = 0.04), and consultant ophthalmologists (100% vs. 83%, *P* = 0.006), but not to ocular oncologists (100% vs. 98%; *P* > 0.99, Bonferroni-corrected *P* values). However, there were no significant differences in specificity between the AI and resident ophthalmologists (74% vs. 63%; *P* = 0.08), consultant ophthalmologists (74% vs. 78%; *P* = 0.36), and ocular oncologists (74% vs. 70%; *P* > 0.99, Bonferroni-corrected *P* values). **B,** When aggregating the data from all ophthalmologist categories and comparing it to the algorithm, the algorithm demonstrated superior sensitivity (Mann−Whitney *U P* = 0.006) but no significant difference in specificity (*P* = 0.54).

Post hoc pairwise comparisons using Dunn's test revealed that the algorithm exhibited significantly higher sensitivity compared with that of resident ophthalmologists (100% vs 85%; *P* = 0.04), and consultant ophthalmologists (100% vs. 83%; *P* = 0.006) but not ocular oncologists (100% vs. 98%; *P* > 0.99, Bonferroni-corrected *P* values; Fig 3A).

There was no significant difference in sensitivity between the 3 categories of ophthalmologists (resident ophthalmologists, consultant ophthalmologists, and ocular oncologists, Kruskal–Wallis χ^2^ = 3.6, df = 2, *P* = 0.16).

### Test Cohort Analysis of Specificity

The Kruskal–Wallis test did not show a significant difference in specificities across the 4 observer categories (algorithm, resident ophthalmologists, consultant ophthalmologists, and ocular oncologists; χ^2^ = 4.0; *P* = 0.26), with post hoc pairwise comparisons using Dunn's test revealing similar specificity to that of resident ophthalmologists (74% vs. 63%; *P* = 0.08), consultant ophthalmologists (74% vs. 78%; *P* = 0.36), and ocular oncologists (74% vs. 70%; *P* > 0.99, Bonferroni-corrected *P* values; Fig 3A).

There was no significant difference in specificity between the 3 categories of ophthalmologists (resident ophthalmologists, consultant ophthalmologists, and ocular oncologists; Kruskal–Wallis χ^2^: 2.5; *P* = 0.29).

### Test Cohort Comparison between all Ophthalmologists and the Algorithm

To compare the collective performance of ophthalmologists with that of the algorithm, the Mann–Whitney *U* test was employed, considering both sensitivity and specificity measures. The outcomes indicated that the algorithm had higher sensitivity (*P* = 0.006) but not specificity (*P* = 0.54) compared with the aggregated data from ophthalmologists (illustrated in Fig 3B). Similarly, when excluding ocular oncologists, the algorithm had higher sensitivity (*P* < 0.001) but not specificity (*P* = 0.37).

### Algorithm Performance on Wide Field and Standard Field Photographs in the Test Cohort

We assessed the algorithm's performance separately for the 31 wide field and 55 standard field fundus photographs in the test cohort to determine if the performance varied between image types, despite the algorithm being trained on both. The findings indicate comparable performance of the algorithm across both types of images. For wide field photographs, the algorithm achieved an AUC of 0.89 (95% confidence interval [CI] 0.76–1.00), with a Gini index of 0.88 and F-score of 0.87. Sensitivity and specificity were 100% and 62%, respectively. For standard field photographs, the algorithm achieved an identical AUC of 0.89 (95% CI, 0.81–0.97), with a Gini index of 0.78 and an F-score of 0.67. Sensitivity and specificity for standard field photographs were 100% and 77%, respectively.

### Test Cohort Algorithm versus MOLES and TFSOM-UHHD

**Figure 4:**
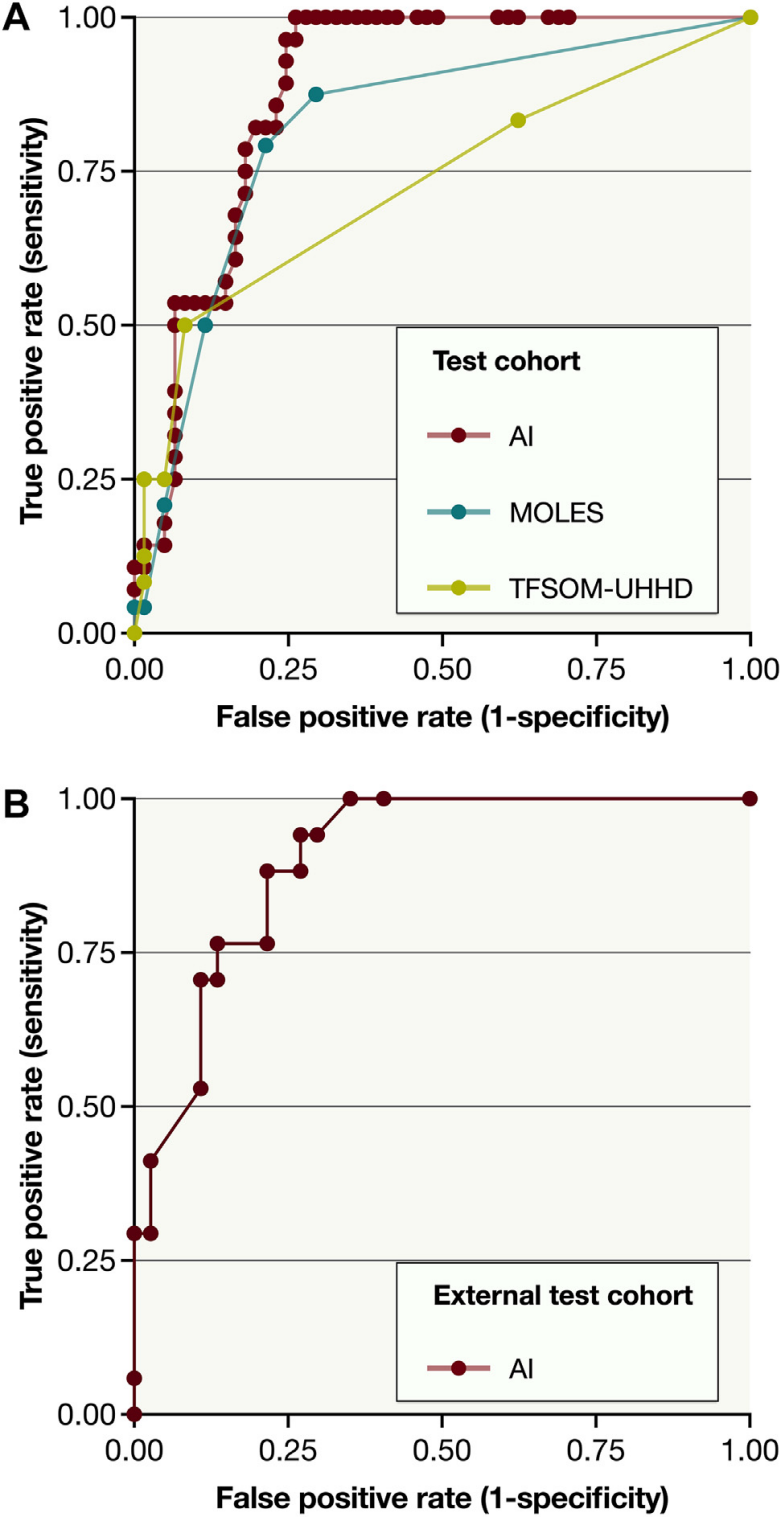
Receiver operating characteristics of the algorithm (AI) in the test and external test cohorts. **A,** AI versus the MOLES (Mushroom shape, Orange pigment, Large size, Enlarging tumor, and Subretinal fluid) and the TFSOM-UHHD (“To Find Small Ocular Melanoma Using Helpful Hints Daily”) mnemonics in the test cohort (n = 86). Unlike the AI, which solely analyzed the photographs, MOLES and TFSOM-UHHD scores incorporates additional data from ultrasonography and/or OCT to ascertain the presence of risk factors (e.g., ultrasonographic hollowness and subretinal fluid). In a pairwise DeLong’s test, the AUC for the AI (0.88; 95% CI, 0.82–0.95) was significantly higher than the AUC for MOLES (0.77; 95% CI, 0.66–0.88; *P* < 0.001) and TFSOM-UHHD (0.67; 95% CI, 0.54–0.81; *P* < 0.001, Bonferroni-corrected *P* values). **B,** AI performance in the external test cohort (n = 53), which consisted exclusively of wide and standard field fundus photographs taken from centers outside of St. Erik Eye Hospital. Again, the algorithm achieved an AUC of 0.88 (95% CI, 0.74–1.00). AUC = area under the curve; MOLES = Mushroom shape, Orange pigment, Large size, Enlargement, and Subretinal fluid; TFSOM-UHHD = To Find Small Ocular Melanoma Using Helpful Hints Daily.

We compared the receiver operating characteristics of the algorithm versus scores determined by ophthalmologists using the MOLES and TFSOM-UHHD classifications on the test set of 86 images. Unlike the algorithm, which solely analyzed the photographs, the MOLES and TFSOM-UHHD scores incorporated additional data from ultrasonography and/or OCT to ascertain the presence of risk factors (e.g., ultrasonographic hollowness and subretinal fluid, as described in medical records after examinations by ocular oncologists). Despite this, the algorithm achieved an AUC of 0.88 (95% CI, 0.82–0.95, Gini index: 0.75, F-score: 0.72), which compares favorably to the AUC for MOLES at 0.77 (95% CI, 0.66–0.88; Gini index: 0.54; F-score using a MOLES score of ≥3 as indicative of melanoma, 0.56), and TFSOM-UHHD at 0.67 (95% CI 0.54–0.81; Gini index: 0.34). In a pairwise DeLong’s test, the AUC for the algorithm was significantly higher than the AUC for MOLES (*P* < 0.001) and TFSOM-UHHD (*P* < 0.001, Bonferroni-corrected *P* values; Fig 4A). These findings underscore the algorithm's robustness in lesion classification, even with access to more limited data compared with traditional classification systems.

### External Test Cohort

In the external test cohort of 53 wide field and standard field photos taken exclusively outside St. Erik Eye Hospital, the algorithm achieved an AUC identical to that in the test cohort, at 0.88 (95% CI 0.74–1.00, Gini index 0.77). At the 0.63 decision threshold, the sensitivity and specificity were 80% and 81%, respectively (F-score 0.71, Fig 4B).

## Discussion

### Main Findings

In this study, we have developed and validated a deep learning algorithm designed to differentiate between small choroidal melanomas and nevi in wide field and standard field fundus photographs. These lesions were initially diagnosed by ocular oncologists who utilized comprehensive diagnostic tools including imaging, biomicroscopy, and ultrasonography during clinical examinations. To mitigate the potential for misclassifications by the ocular oncologists, lesions diagnosed as nevi were subjected to a minimum 5-year follow-up period, allowing for the identification of any lesions initially classified as nevi that may have progressed to small melanomas over time.

The algorithm demonstrated superior sensitivity in distinguishing small choroidal melanomas from nevi compared with both resident and consultant ophthalmologists, while achieving similar sensitivity and specificity to that of ocular oncologists. It outperformed the discriminatory performance of ophthalmologists who used the MOLES and TFSOM-UHHD classifications, even though they had access to additional results of ultrasound and OCT examinations. Moreover, it provided identical assessments for identical images, meaning it will give the same score every time the same image is analyzed, which is not necessarily the case for human observers, who may battle intraobserver and interobserver variability.

### Context

Choroidal melanocytic lesions are common, but melanomas are rare. Small lesions are often discovered in routine examinations for unrelated conditions, such as cataract surgery, diabetic retinopathy screening, and general eye examinations conducted by optometrists. This presents a significant challenge for many healthcare systems, as the availability of subspecialized ocular oncologists is typically limited, and resources must be prioritized for patients with confirmed malignancies rather than for those with potentially benign lesions. Unlike pigmented lesions on the skin, patients cannot monitor choroidal lesions themselves. Consequently, a large number of lesions, of which only a minimal fraction are harmful, must be evaluated by personnel with limited expertise in ocular oncology.

In this context, an algorithm with equivalent performance to ocular oncologists, applicable to both wide and standard field fundus photography, could prove invaluable. We propose that such an algorithm may serve as a valuable tool to aid both ocular oncologists and other healthcare providers in assessing pigmented choroidal lesions. It could be utilized to identify which small lesions should be referred to ocular oncologists, as well as to provide an objective basis for lesion evaluations (e.g., referrals could be indicated for specific scores) or if changes in these scores are observed over time. Furthermore, given its consistent evaluation of identical photographs, the algorithm may facilitate more reliable assessments by experts, particularly in detecting small melanomas with high sensitivity.

### Strengths and Limitations

In the development and validation of our algorithm, we relied heavily on the diagnostic conclusions drawn by ocular oncologists. These specialists had access to an extensive array of diagnostic tools, including imaging facilities, biomicroscopes, and ultrasound equipment. To reduce the risk of misclassification (e.g., incorrectly diagnosing a small lesion as a nevus when it might actually be an early stage melanoma) we mandated a minimum event-free observation period of 5 years for lesions categorized as nevi. However, our algorithm's accuracy is contingent upon the precision of the ocular oncologists' clinical evaluations, particularly for lesions identified as melanoma that received treatment. It is important to note that all Swedish choroidal melanoma diagnoses are centralized to our institution, meaning that lesions that were classified as nevi by us were not diagnosed as melanoma elsewhere. Furthermore, numerous publications corroborate that our incidence rates align with those in similar populations, providing reassurance that our diagnostic standards are representative.^18^, ^19^, ^20^, ^21^, ^22^

Secondly, variability in the evaluation of small choroidal melanocytic lesions by human observers is an inescapable reality, and even ophthalmologists with a subspecialty in ocular oncology are not immune to such variability. In our comparison with the algorithm's performance, only 3 ocular oncologists were engaged. Consequently, the sensitivity and specificity derived from their evaluations are based on a limited observer pool, which may not necessarily reflect the broader ocular oncology community. Although this subspecialty is notably scarce, and these 3 professionals account for over half of the country's ocular oncologists, caution is warranted when generalizing their assessments. It is plausible that their tendency toward higher sensitivity and lower specificity could stem from their routine access to a broad array of diagnostic tools, leading to a more inclusive approach in the assessment of photographs to ensure a thorough examination in clinical settings, thereby minimizing the risk of overlooking potential melanomas.

Thirdly, some may prefer an end-to-end model with image input and classification output over our approach to segment the lesion before classification. This approach was considered and tested in the planning phase of this project. However, it was not feasible to train a model in this manner, given the amount of data we have. The model did not perform adequately and failed to meet our standards. Therefore, comparing our method to such a model would not be meaningful, because the performance would be significantly inferior. Additionally, there are numerous other approaches one might test, such as conventional machine learning models or different network architectures. Pursuing these comparisons would transform our work into a purely machine learning-focused article, which was not our intention. Our approach has distinct advantages. By providing segmented input, we guide the model to focus specifically on the lesion, eliminating the need for the model to learn the spatial localization of melanomas. This approach enhances interpretability and potentially improves classification accuracy by reducing the noise and complexity associated with full-image input. Furthermore, segmentation allows clinicians to visually confirm the areas of interest, fostering better understanding and trust in the model's predictions.

Fourthly, the sample size utilized for the development, validation, and testing of the algorithm was limited, which is a notable consideration given that developers of neural networks generally aim for substantially larger datasets. This limitation suggests the potential for enhanced algorithmic performance with access to a larger and more diverse set of training images. Conversely, its efficacy might diminish for lesions with atypical appearances not represented in the training dataset. Should the validation and testing have involved larger samples, the sensitivity and specificity metrics might have varied. Moving forward, we plan to update and refine the algorithm by training it on expanded datasets and validating its applicability across additional patient cohorts.

Fifthly, a diverse range of cameras and software is employed in the acquisition and analysis of fundus images. Our algorithm's training was limited to images from only 2 types of imaging systems, leaving its effectiveness on alternative platforms untested. Optos ultra-widefield photographs are pseudo colored and may not reproduce true colors accurately. We intentionally mixed wide field and standard field photographs in order to develop an algorithm that was insensitive to deviations from true color, ensuring its applicability to both imaging modalities. However, we advise against its application without prior adaptation and revalidation on other types of images. We excluded low-quality photographs (e.g., issues with focus, movement artifacts, overexposure or underexposure, reflections), which could have introduced a bias favoring images from centers more experienced in examining fundus lesions and disadvantaging smaller centers. This could potentially reduce the algorithm's usability in such settings. However, we did not observe lower image quality in the external test cohort, and the algorithm's performance remained consistent. Additionally, because lesions obscured by vitreous bleeding, retinal detachment, or similar conditions were excluded, our algorithm was not trained on such images, which may lead to misclassification. Nevertheless, this might not be a major issue in clinical practice, as fundus photographs should not be the primary diagnostic modality for obscured lesions, regardless of whether a deep learning algorithm is used.

We have developed and validated a deep learning algorithm that demonstrates high sensitivity and specificity for separating small choroidal melanomas from nevi. This algorithm matched or surpassed the diagnostic capabilities of human ophthalmologists who examined the same fundus photographs. Further investigation is needed, ideally in prospective cohorts, to validate the practical application of this algorithm in clinical settings and to assess whether its use can enhance patient care and outcomes compared with traditional ophthalmologic evaluations.

## Data Sharing Statement

Due to the sensitive nature of the clinical data, including images utilized in this study, the authors are unable to share these materials in compliance with Swedish law. The confidentiality and privacy regulations governing patient information strictly prohibit the distribution of such data.
