# Supplementary material for: Development and Validation of a Deep Learning Algorithm for Differentiation of Choroidal Nevi from Small Melanoma in Fundus Photographs
Source: Ophthalmol Sci. 2024 Aug 30;5(1):100613. doi: 10.1016/j.xops.2024.100613 (PMC11483474; doi:10.1016/j.xops.2024.100613)
Supplement: Supplementary File [file mmc1.docx]

Deep learning algorithms have enhanced the accuracy and reproducibility of image-based diagnoses across various ocular diseases. Differentiating small choroidal melanomas from nevi, a common yet challenging task, could benefit from improved diagnostic tools.

**Objective:**
To develop and validate a deep learning algorithm capable of differentiating small choroidal melanomas from nevi.

**Design, Setting, and Participants:**
Wide- and standard field fundus photographs from patients diagnosed with choroidal nevi or melanoma were collected across multiple centers. Diagnoses had been established by ocular oncologists in clinical examinations, using a comprehensive array of diagnostic tools. To be classified as a nevus, a lesion had to be followed for at least 5 years without being re-diagnosed as a melanoma. We trained and validated a neural network optimized for image classification across cohorts of 495 and 168 images, subsequently testing it on a separate set of 89 images.

**Exposure:**
Algorithm trained via deep learning.

**Main Outcomes and Measures:**
Sensitivity and specificity of the deep learning algorithm in differentiation of small choroidal melanomas from nevi.

**Results:**
In testing, the algorithm achieved 100% sensitivity in identifying small choroidal melanomas from nevi, with a specificity rate of 74%, using an optimal operating point of 0.63 (on a scale from 0.00 to 1.00) determined from independent training and validation datasets. It outperformed 12 ophthalmologists in sensitivity (Mann-Whitney *U* *P*=0.006) but not specificity (*P*=0.54). When comparing by level of experience, the algorithm showed higher sensitivity than both resident and consultant ophthalmologists (Dunn's test *P*=0.04 and *P*=0.006, respectively) but not ocular oncologists (*P*>0.99). Furthermore, the algorithm demonstrated greater discriminative capacity than ophthalmologists who used the MOLES and TFSOM-UHHD risk factors (DeLong’s test *P*<0.001, all *P* values Bonferroni corrected), despite the latter having access to supplementary examination data from ultrasonography and optical coherence tomography (OCT).

**Conclusions and Relevance:**
This study develops and validates a deep learning algorithm for differentiating small choroidal melanomas from nevi, that matches or surpasses the discriminatory performance of experienced human ophthalmologists. Further research will aim to validate its utility in clinical settin
